# Supplementary material for: An analysis of the accuracy of retrospective birth location recall using sibling data
Source: Nat Commun. 2024 Mar 26;15:2665. doi: 10.1038/s41467-024-46781-z (PMC10965954; doi:10.1038/s41467-024-46781-z)
Supplement: Supplementary file 1 — Supplementary Information [file 41467_2024_46781_MOESM1_ESM.pdf]

Supplementary Information:

An analysis of the accuracy of retrospective birth location recall using sibling data

Stephanie von Hinke[1,2,3,*] and Nicolai Vitt[1,*]

[1]School of Economics, University of Bristol, Bristol, United Kingdom
[2]Institute for Fiscal Studies, London, United Kingdom
[3]Institute for the Study of Labor (IZA), Bonn, Germany
[*]Corresponding authors. E-mail: s.vonhinke@bristol.ac.uk, nicolai.vitt@bristol.ac.uk

Contents

Supplementary Note 1: Studies using retrospective birth location data in the UK Biobank **2**

Supplementary Background 1: Birth location processing in the UK Biobank **4**

Supplementary Results 1: Sensitivity analysis **5**

Supplementary Results 2: Heterogeneity analysis **6**

Supplementary Results 3: Analysis of repeat birth location reports **14**

Supplementary Results 4: Attenuation bias **15**

Supplementary Results 5: Bias in analyses controlling for district of birth fixed effects **17**

Supplementary Results 6: Spatial distribution of genetic principal components **18**

Supplementary Analysis 1: Phenotype comparisons in Abdellaoui et al. (2019) **19**

Supplementary Results 7: Descriptive statistics **22**

Supplementary Note 2: Attenuation bias - Classical measurement error model **24**

Supplementary Results 8: Distribution of measurement error **25**

Supplementary References **28**

Supplementary Note 1: Studies using retrospective birth location data in the UK Biobank

In this appendix, we summarize a number of high-profile studies that use the retrospective birth location data in the UK Biobank.

Abdellaoui et al. (2019)[1] use data on birth location and current residential location in the UK Biobank to study genetic consequences of social stratification. They show substantial geographic clustering in polygenic indices for various traits (even after controlling for ancestry) across 378 local authorities in the UK. Significant differences in phenotypes and polygenic indices are found between former coal mining areas and the rest of the UK, with e.g. lower educational attainment and lower PGIs for education observed in former coal mining regions. Furthermore, individuals migrating out of coal mining regions were found to have more favourable phenotypes and polygenic indices than those staying. Their findings suggest that recent internal migration within Great Britain driven by socioeconomic factors has led to genetic as well as social stratification. We replicate some of the analysis of this study in Supplementary Analysis 1.

Haworth et al. (2019)[2] analyse associations between birth location coordinates in the UK Biobank and genetic variation. They illustrate that accounting for interview location and genetic principal components is not sufficient to adjust for stratification. Polygenic indices for various complex traits are shown to be related to birth coordinates, and a GWAS of birth coordinates yields significant findings. With phenotypes also being geographically structured, these findings illustrate the potential for biased associations between genetic variation and phenotypic outcomes.

Howe et al. (2019)[3] use Mendelian randomization to study assortative mating based on alcohol consumption. The study finds that a genetic variant robustly associated with alcohol consumption not only increases an individual's own alcohol intake, but also the alcohol intake of their spouse. Spouses' genotypes are furthermore found to be positively correlated, providing evidence of a positive relationship in spouses' alcohol consumption prior to the start of their relationship. The study uses birth coordinates (in addition to genetic principal components) to explore the relevance of population stratification in explaining the genetic and phenotypic correlations within spouse pairs. In a sensitivity analysis, they limit the sample to spouses born within 100 km of each other.

Von Hinke and Sorensen (2023)[4] study the long-term effects of exposure to the London smog during the in-utero and early childhood periods on later-life health and human capital. They use the information on participants' district and date of birth in the UK Biobank to capture whether (and during which period of pregnancy or childhood) they were exposed to a 5-day period of extremely high pollution in London during

1952. Their findings suggest that exposure to air pollution lead to a long-term reduction in fluid intelligence and worse respiratory health.

Supplementary Background 1: Birth location processing in the UK Biobank

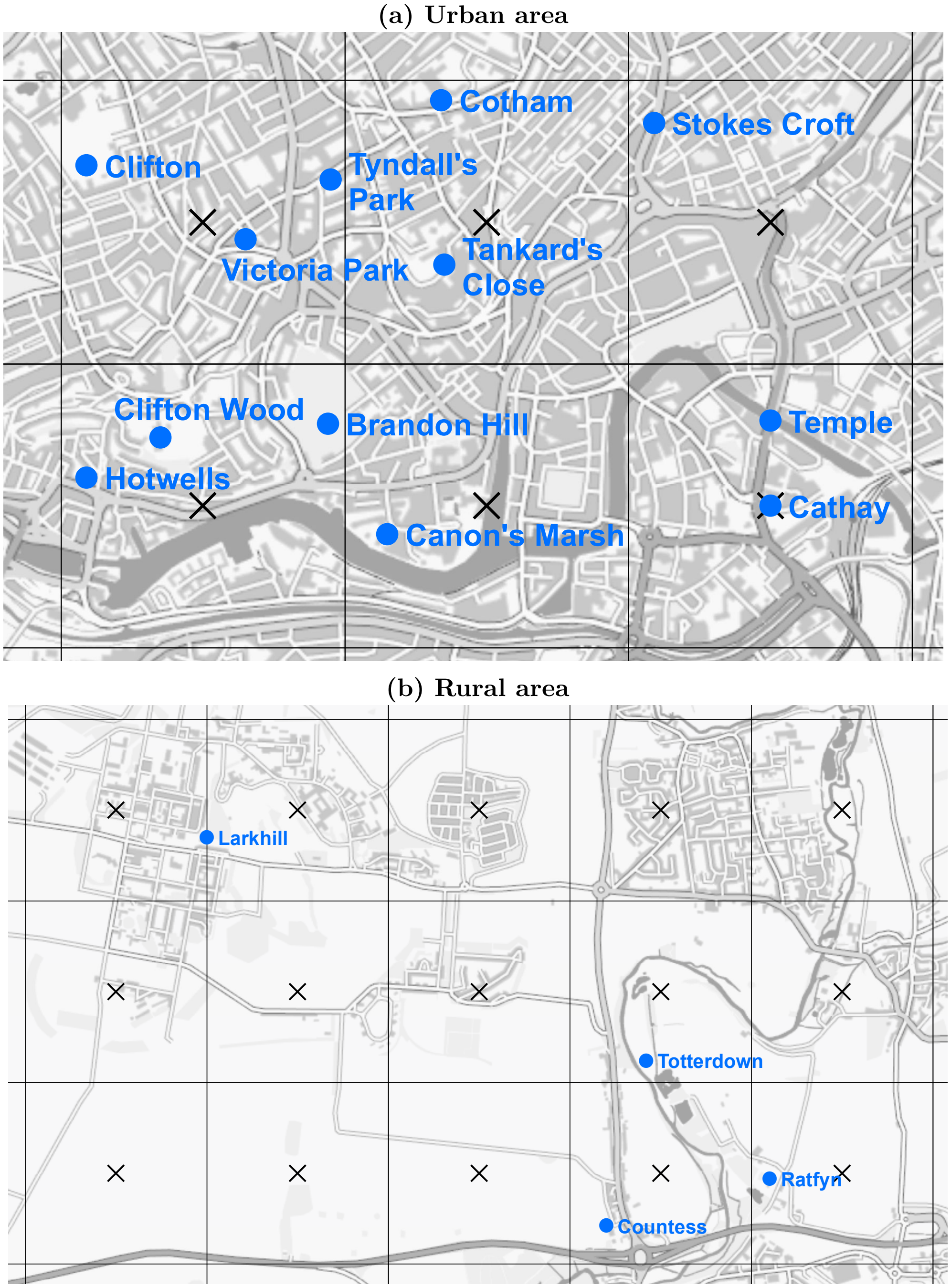

Supplementary Figure 1: Examples of potential birth locations and the corresponding rounded grid coordinates in the UK Biobank. Panel **(a)** shows an example of an urban area, panel **(b)** an example of a rural area. Blue points are the locations in the interviewer list for the UKB birth location question. Grid squares are 1km by 1km areas with the same rounded birth coordinates, the corresponding rounded coordinates are marked with an '×'. Note that some of the birth coordinates observed in the UK Biobank data do not correspond to locations on the interviewer list, possibly corresponding to answers given/recorded at a lower level of detail (e.g. city name rather than neighbourhood). Contains OS data licensed under the Open Government Licence v3.0 © Crown Copyright and Database Right 2020.

Supplementary Results 1: Sensitivity analysis

Supplementary Table 1: Differences in siblings' birth location and their age gap - nonlinear specification

	Different birth location:							
	(1)	(2)	(3)	(4)	(5)	(6)	(7)	(8)
	Parish	District	County	d>0km	d>5km	d>10km	d>30km	d>50km
**Regression coefficients:**								
Age gap (years)	0.0153	0.0138	0.0112	0.0196	0.0182	0.0134	0.0139	0.0103
	[0.0089,0.0217]	[0.0074,0.0201]	[0.0061,0.0163]	[0.0130,0.0261]	[0.0120,0.0245]	[0.0079,0.0188]	[0.0093,0.0185]	[0.0060,0.0146]
Age gap squared	-0.0004	-0.0004	-0.0003	-0.0006	-0.0006	-0.0005	-0.0005	-0.0003
	[-0.0009,0.0000]	[-0.0008,0.0001]	[-0.0007,0.0001]	[-0.0010,-0.0001]	[-0.0010,-0.0002]	[-0.0008,-0.0001]	[-0.0009,-0.0002]	[-0.0006,0.0000]
Constant	0.2936	0.2806	0.1136	0.4688	0.2350	0.1451	0.0692	0.0566
	[0.2755,0.3117]	[0.2627,0.2985]	[0.0999,0.1272]	[0.4498,0.4877]	[0.2178,0.2523]	[0.1303,0.1599]	[0.0572,0.0811]	[0.0455,0.0677]
**Derived probabilities:**								
$\hat{q}(agegap = 1)$	0.0149	0.0134	0.0109	0.0190	0.0176	0.0129	0.0134	0.0100
	[0.0089,0.0209]	[0.0075,0.0194]	[0.0061,0.0157]	[0.0129,0.0252]	[0.0118,0.0234]	[0.0079,0.0180]	[0.0091,0.0177]	[0.0060,0.0140]
$\hat{q}(agegap = 3)$	0.0140	0.0127	0.0103	0.0179	0.0164	0.0120	0.0123	0.0094
	[0.0088,0.0192]	[0.0076,0.0178]	[0.0062,0.0144]	[0.0126,0.0232]	[0.0114,0.0214]	[0.0077,0.0164]	[0.0087,0.0160]	[0.0060,0.0128]
$\hat{q}(agegap = 6)$	0.0127	0.0116	0.0093	0.0162	0.0145	0.0107	0.0108	0.0085
	[0.0087,0.0168]	[0.0077,0.0156]	[0.0062,0.0125]	[0.0121,0.0204]	[0.0107,0.0184]	[0.0073,0.0140]	[0.0080,0.0136]	[0.0059,0.0111]
$\hat{q}(agegap = 9)$	0.0115	0.0106	0.0084	0.0146	0.0127	0.0093	0.0092	0.0076
	[0.0085,0.0145]	[0.0076,0.0135]	[0.0061,0.0107]	[0.0114,0.0177]	[0.0098,0.0156]	[0.0069,0.0118]	[0.0072,0.0113]	[0.0057,0.0095]
$\hat{p}$	0.1595	0.1518	0.0585	0.2711	0.1254	0.0754	0.0352	0.0287
	[0.1488,0.1703]	[0.1413,0.1624]	[0.0512,0.0657]	[0.2581,0.2841]	[0.1155,0.1352]	[0.0674,0.0834]	[0.0290,0.0414]	[0.0230,0.0344]
N (sibling pairs)	18,479	18,479	18,479	18,479	18,479	18,479	18,479	18,479

Notes: $\hat{q}$ is the estimated increase in the probability of at least one house move when increasing the age gap between siblings by 1 additional year, it is given above for different age gap levels. $\hat{p}$ is the estimated probability of an error in the recorded birth location of a UKB participant. The sample is restricted to the first sibling pair observed in each family. 95% confidence intervals based on heteroskedasticity robust standard errors are shown in brackets. Standard errors for $\hat{q}$ and $\hat{p}$ were computed using the delta method.

Supplementary Results 2: Heterogeneity analysis

In this Appendix we explore heterogeneities in the annual probability of a household move and the probability of measurement error along several dimensions. Supplementary Table 2 shows limited birth cohort differences in the probability of household moves to a different district, but a larger probability of measurement error among later (i.e. younger) cohorts. The sex composition of the sibling pairs is, as expected, not found to matter for the probabilities of household moves and measurement error (Supplementary Table 3).

Supplementary Table 4 shows a substantially higher probability of moving out of rural districts at 2.1% annually, compared to 0.8% and 0.9% for urban/municipal and metropolitan districts respectively. A cross-tabulation of district types reported by the 1st (i.e. older) and 2nd (i.e. younger) sibling in Supplementary Table 5 suggests that a large share of these moves originating in rural districts was to urban / municipal districts. The probability of measurement error also differs substantially by district type, with Supplementary Table 4 showing this is 13.8% for sibling pairs with the older sibling born in urban / municipal districts, 28.2% for rural districts and 39.4% for metropolitan districts.

Supplementary Table 6 provides additional evidence based on an alternative approximation of urbanicity, comparing population density quartiles of the older sibling's district of birth. Again, we find a higher probability of moving out of more rural areas, with an annual probability of 1.4% for the lowest population density quartile compared to 0.6% for the highest density quartile. The probability of measurement error is also found to be largest in less densely populated areas.

In Supplementary Table 7 we examine heterogeneity between areas with high and low levels of educational attainment, according to data from the 1951 census[5,6]. Mobility is higher among families living in more highly educated areas (i.e., districts with a low share of adults who left education by age 14), with an annual move probability of 1.4% in districts with the highest levels of education compared to 0.7% in districts with the lowest education levels. For the probability of measurement error, the picture is less clear but suggestive of higher error probabilities in more highly educated areas.

In Supplementary Table 8, we use the average polygenic index (PGI) for education of the two siblings to proxy for the parental PGI and examine whether mobility and misreporting are heterogeneous between families with higher and lower polygenic indices for education. The PGI is the best linear genetic predictor of education, but does not necessarily capture biological or non-modifiable effects. There is no systematic heterogeneity in the move probability, but some suggestion – perhaps unexpectedly – that error probabilities are slightly higher among families with a higher genetic propensity for education.

Previous research shows selective migration based on one's PGI[1]. More specifically, they show that individuals who leave UK coal mining areas carry more education-increasing alleles compared to individuals elsewhere

in the UK, highlighting genetic correlates of social stratification. We explore this in an alternative way, examining whether a family's genetic propensity for education differentially affects mobility in areas with low and high levels of education. Supplementary Table 9 indeed shows a slightly higher probability of moving for those with above-median PGIs, but in contrast to previous research, we find larger move probabilities in higher educated areas. This contrast in findings may be explained by the fact that we are examining mobility in the 1940-1970s among families with young children, while previous findings[1] likely capture mobility at a later time (e.g. following the closure of UK coal mines in the 1980s) during early adulthood of the UKB participants.

In addition to characteristics of the UKB participants and their area of birth, measurement error may also be related to the UKB assessment centre at which they were first interviewed. The birth location data is based on an interaction between the participant and an interviewer, who identified the place of birth from a long list of place names in the computer system using the information given by the participant. Thus the accuracy of the recorded birth location may be subject to interviewer effects. Similarly, any errors in processing reported locations of birth to grid coordinates by the data providers may cause measurement error. For example, changes in regions' boundaries may lead one sibling to report a different location from the other sibling as their area of birth was known under a different name, even though they did not move. Hence, variation in measurement error by assessment centre or region of birth may be driven by interviewer effects or processing errors. Although we cannot distinguish between the two, we explore their (joint) importance by studying heterogeneity in the error probability across the 22 assessment centres of the UKB as well as the 12 regions of birth that we observe. Panel (a) of Supplementary Figure 2 shows substantial heterogeneity in the discordance share for siblings' district of birth: only 26% of participants interviewed in Edinburgh are recorded to have a different district of birth to their sibling, whilst this is 55% of those interviewed in Barts. Naturally, these differences translate to substantial heterogeneity in the estimated probability of measurement error shown in panel (b), from 11% in Edinburgh to 30% in Barts. In line with this, we find a low error probability for siblings born in Scotland and one of the highest in the London area (see Supplementary Figure 3).

Supplementary Table 2: Differences in siblings' birth district and their age gap - across different birth cohorts

	Different birth district:				
	(1) 1939-44	(2) 1945-49	(3) 1950-54	(4) 1955-59	(5) 1960-68
**Regression coefficients:**					
Age gap (years)	0.012	0.008	0.011	0.013	0.010
	[0.008,0.016]	[0.004,0.012]	[0.006,0.017]	[0.005,0.021]	[-0.006,0.025]
Constant	0.253	0.287	0.291	0.315	0.331
	[0.232,0.274]	[0.265,0.308]	[0.262,0.319]	[0.278,0.351]	[0.283,0.378]
**Derived probabilities:**					
$\hat{q}$ (move probability)	0.012	0.008	0.011	0.013	0.010
	[0.008,0.016]	[0.004,0.012]	[0.006,0.017]	[0.005,0.021]	[-0.006,0.025]
$\hat{p}$ (error probability)	0.136	0.155	0.158	0.172	0.182
	[0.124,0.148]	[0.142,0.168]	[0.141,0.175]	[0.150,0.194]	[0.153,0.211]
N (sibling pairs)	5,745	4,950	3,282	2,416	1,711

Notes: The sample is restricted to the first sibling pair observed in each family. Each column of the table restricts the sample to sibling pairs in which the first (i.e. older) sibling is born in the given range of years. 95% confidence intervals based on heteroskedasticity robust standard errors are shown in brackets. Standard errors for $\hat{p}$ were computed using the delta method.

Supplementary Table 3: Differences in siblings' birth district and their age gap - across sibling sex compositions

	Gender composition of sibling pair:		
	(1) F/F	(2) F/M	(3) M/M
**Regression coefficients:**			
Age gap (years)	0.008	0.009	0.008
	[0.005,0.012]	[0.006,0.013]	[0.003,0.013]
Constant	0.285	0.297	0.293
	[0.266,0.305]	[0.280,0.315]	[0.266,0.319]
**Derived probabilities:**			
$\hat{q}$ (move probability)	0.008	0.009	0.008
	[0.005,0.012]	[0.006,0.013]	[0.003,0.013]
$\hat{p}$ (error probability)	0.154	0.162	0.159
	[0.143,0.166]	[0.151,0.172]	[0.143,0.175]
N (sibling pairs)	6,506	8,342	3,631

Notes: The sample is restricted to the first sibling pair observed in each family. Column (1) restricts the sample to female sibling pairs, (2) to mixed-sex sibling pairs, (3) to male sibling pairs. 95% confidence intervals based on heteroskedasticity robust standard errors are shown in brackets. Standard errors for $\hat{p}$ were computed using the delta method.

Supplementary Table 4: Differences in siblings' birth district and their age gap - across different district types

	Different birth district:		
	(1) Rural	(2) Urban / municipal	(3) Metropolitan
**Regression coefficients:**			
Age gap (years)	0.022	0.008	0.009
	[0.015,0.029]	[0.006,0.010]	[-0.003,0.022]
Constant	0.479	0.257	0.632
	[0.439,0.520]	[0.245,0.270]	[0.564,0.701]
**Derived probabilities:**			
$\hat{q}$ (move probability)	0.022	0.008	0.009
	[0.015,0.029]	[0.006,0.010]	[-0.003,0.022]
$\hat{p}$ (error probability)	0.278	0.138	0.394
	[0.250,0.306]	[0.131,0.146]	[0.337,0.450]
N (sibling pairs)	1,725	14,621	563

Notes: The sample is restricted to the first sibling pair observed in each family. Each column of the table restricts the sample to sibling pairs in which the first (i.e. older) sibling is born in a district of the given type. 95% confidence intervals based on heteroskedasticity robust standard errors are shown in brackets. Standard errors for $\hat{p}$ were computed using the delta method.

Supplementary Table 5: Differences in siblings' birth district - by district type

	**2nd sibling:**				
	Same district	Different district			Total
**1st sibling:**		Rural	Urban / muncipal	Metropolitan	
Rural	42.63%	8.62%	47.87%	0.87%	100.00%
	(732)	(148)	(822)	(15)	(1,717)
Urban / municipal	70.93%	6.16%	22.08%	0.83%	100.00%
	(10,332)	(897)	(3,216)	(121)	(14,566)
Metropolitan	33.03%	4.67%	35.19%	27.11%	100.00%
	(184)	(26)	(196)	(151)	(557)
Total	66.79%	6.36%	25.14%	1.70%	100.00%
	(11,248)	(1,071)	(4,234)	(287)	(16,840)

Notes: The table shows row percentages and the corresponding frequency counts in parentheses.

Supplementary Table 6: Differences in siblings' birth district and their age gap - across quartiles of the district population density

	Quartiles of district-level population density:			
	(1) Q1	(2) Q2	(3) Q3	(4) Q4
**Regression coefficients:**				
Age gap (years)	0.014	0.012	0.007	0.004
	[0.009,0.019]	[0.007,0.016]	[0.002,0.011]	[0.001,0.008]
Constant	0.375	0.358	0.231	0.218
	[0.349,0.400]	[0.333,0.382]	[0.208,0.255]	[0.199,0.237]
**Derived probabilities:**				
$\hat{q}$ (move probability)	0.014	0.012	0.007	0.004
	[0.009,0.019]	[0.007,0.016]	[0.002,0.011]	[0.001,0.008]
$\hat{p}$ (error probability)	0.209	0.199	0.123	0.116
	[0.193,0.226]	[0.183,0.214]	[0.110,0.137]	[0.105,0.126]
N (sibling pairs)	4,262	4,638	3,889	5,690

Notes: The sample is restricted to the first sibling pair observed in each family. Each column of the table restricts the sample to sibling pairs in which the first (i.e. older) sibling is born in a district from the given population density quartile. Q1 denotes the 25% of districts with the lowest population density, Q4 the 25% of districts with the highest population density. 95% confidence intervals based on heteroskedasticity robust standard errors are shown in brackets. Standard errors for $\hat{p}$ were computed using the delta method.

Supplementary Table 7: Differences in siblings' birth district and their age gap - across quartiles of the district share of early school leavers

	Quartiles of share left education by age 14:			
	(1) Q1	(2) Q2	(3) Q3	(4) Q4
**Regression coefficients:**				
Age gap (years)	0.014	0.009	0.010	0.005
	[0.010,0.019]	[0.004,0.013]	[0.005,0.014]	[0.001,0.009]
Constant	0.363	0.264	0.231	0.286
	[0.339,0.387]	[0.239,0.289]	[0.207,0.255]	[0.265,0.307]
**Derived probabilities:**				
$\hat{q}$ (move probability)	0.014	0.009	0.010	0.005
	[0.010,0.019]	[0.004,0.013]	[0.005,0.014]	[0.001,0.009]
$\hat{p}$ (error probability)	0.202	0.142	0.123	0.155
	[0.187,0.217]	[0.128,0.157]	[0.110,0.137]	[0.143,0.167]
N (sibling pairs)	4,934	3,899	3,879	5,767

Notes: The sample is restricted to the first sibling pair observed in each family. Each column of the table restricts the sample to sibling pairs in which the first (i.e. older) sibling is born in a district from the given quartile of the district-level share of those who left full-time education by age 14 according to the 1951 census[5,6]. Q1 denotes the 25% of districts with the lowest share of early school leavers, Q4 the 25% of districts with the highest share of early school leavers. 95% confidence intervals based on heteroskedasticity robust standard errors are shown in brackets. Standard errors for $\hat{p}$ were computed using the delta method.

Supplementary Table 8: Differences in siblings' birth district and their age gap - across quartiles of the polygenic index for education

	Quartiles of PGI for education:			
	(1) Q1	(2) Q2	(3) Q3	(4) Q4
Age gap (years)	0.008	0.007	0.013	0.009
	[0.004,0.012]	[0.003,0.011]	[0.009,0.018]	[0.005,0.014]
Constant	0.254	0.290	0.277	0.333
	[0.231,0.277]	[0.266,0.313]	[0.253,0.300]	[0.308,0.358]
**Derived probabilities:**				
$\hat{q}$ (move probability)	0.008	0.007	0.013	0.009
	[0.004,0.012]	[0.003,0.011]	[0.009,0.018]	[0.005,0.014]
$\hat{p}$ (error probability)	0.136	0.157	0.149	0.183
	[0.123,0.149]	[0.143,0.171]	[0.136,0.163]	[0.168,0.199]
N (sibling pairs)	4,512	4,510	4,512	4,511

Notes: The sample is restricted to the first sibling pair observed in each family. Each column of the table restricts the sample to sibling pairs in which the mean PGI for education of the sibling pair lies in the given quartile of the distribution. Q1 denotes the 25% of sibling pairs with the lowest mean PGI, Q4 the 25% with the highest mean PGI. The PGI for education is from the PGI repository[7]. 95% confidence intervals based on heteroskedasticity robust standard errors are shown in brackets. Standard errors for $\hat{p}$ were computed using the delta method.

Supplementary Table 9: Differences in siblings' birth district and their age gap - by district shares of early school leavers and siblings' polygenic index for education

	Low education area		High education area	
	(1) Low PGI	(2) High PGI	(3) Low PGI	(4) High PGI
Age gap (years)	0.008	0.009	0.009	0.014
	[0.004,0.012]	[0.004,0.013]	[0.004,0.013]	[0.010,0.019]
Constant	0.261	0.268	0.285	0.339
	[0.237,0.284]	[0.242,0.294]	[0.261,0.310]	[0.314,0.364]
**Derived probabilities:**				
$\hat{q}$ (move probability)	0.008	0.009	0.009	0.014
	[0.004,0.012]	[0.004,0.013]	[0.004,0.013]	[0.010,0.019]
$\hat{p}$ (error probability)	0.140	0.144	0.155	0.187
	[0.126,0.154]	[0.129,0.159]	[0.140,0.169]	[0.172,0.203]
N (sibling pairs)	4,286	3,712	3,992	4,564

Notes: The sample is restricted to the first sibling pair observed in each family. Columns (1)-(2) of the table restrict the sample to sibling pairs in which the first (i.e. older) sibling is born in a district with an above-median share of those who left full-time education by age 14 according to the 1951 census[5,6], columns (3)-(4) restrict the sample to sibling pairs in which the first sibling is born in a district with a below-median share of early school leavers. Columns (1) and (3) furthermore restrict the sample to sibling pairs in which the mean PGI for education of the sibling pair is below the median, columns (2) and (4) restrict to sibling pairs with an above-median PGI. The PGI for education is from the PGI repository[7]. 95% confidence intervals based on heteroskedasticity robust standard errors are shown in brackets. Standard errors for $\hat{p}$ were computed using the delta method.

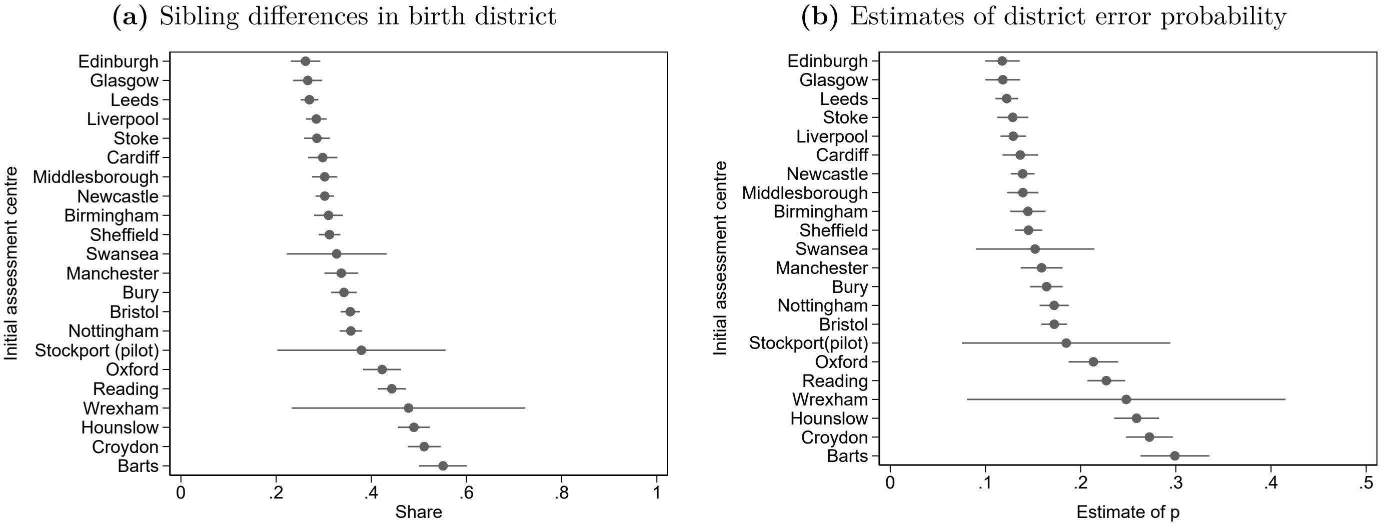

Supplementary Figure 2: Accuracy of siblings' birth district across assessment centre locations. Panel **(a)** shows for each UKB assessment centre the share of individuals in the sibling sample who reported a different birth district to their sibling. Panel **(b)** shows for each UKB assessment centre the derived estimate of the error probability p (with the move probability q jointly estimated across all assessment centres). These analyses were conducted at the individual level (using both siblings in a family) with a sample size of $n = 36,958$ individuals. Horizontal bars represent 95% confidence intervals based on standard errors clustered at the family level. Source data are provided as a source data file.

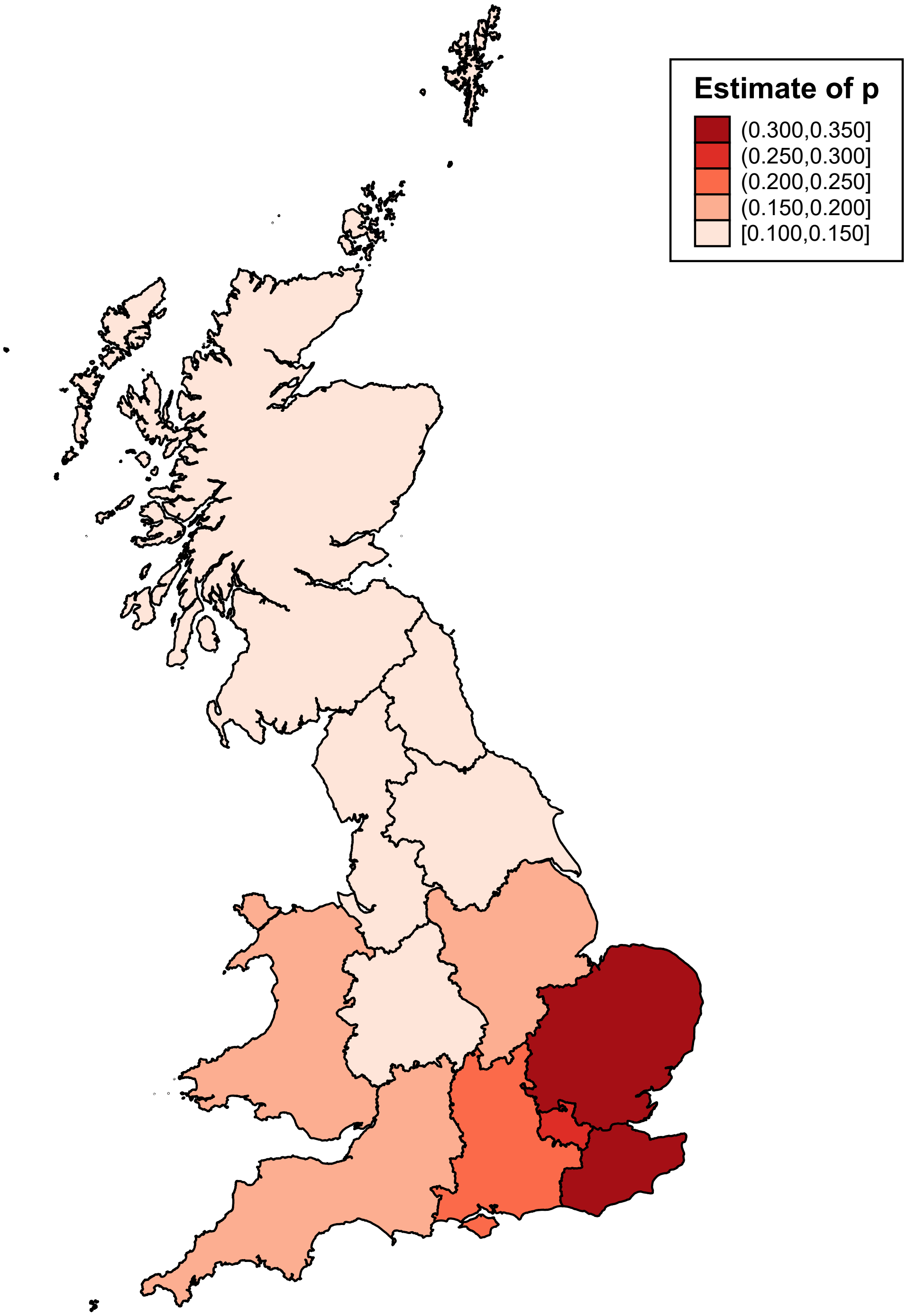

Supplementary Figure 3: Birth district measurement error - across regions of the UK. The map shows the derived estimate of the error probability p for the regions of the UK. The analysis was conducted at the individual level (using both siblings in a family) with a sample size of $n = 36,958$ individuals. q was jointly estimated across all regions. The boundary map is adapted from www.VisionofBritain.org.uk[6] and is copyright of the Great Britain Historical GIS Project and the University of Portsmouth. Source data are provided as a source data file.

Supplementary Results 3: Analysis of repeat birth location reports

Supplementary Table 10: Differences in repeat birth location reports

	Different birth location:							
	(1) Parish	(2) District	(3) County	(4) d>0km	(5) d>5km	(6) d>10km	(7) d>30km	(8) d>50km
Mean	0.279	0.268	0.060	0.431	0.209	0.101	0.037	0.024
	[0.273,0.284]	[0.263,0.274]	[0.057,0.063]	[0.425,0.437]	[0.204,0.214]	[0.098,0.105]	[0.035,0.039]	[0.022,0.026]
$\hat{p}$ (error probability)	0.151	0.145	0.030	0.246	0.111	0.052	0.019	0.012
	[0.147,0.154]	[0.141,0.148]	[0.029,0.032]	[0.242,0.250]	[0.108,0.114]	[0.050,0.054]	[0.017,0.020]	[0.011,0.013]
N	24,838	24,838	24,838	24,838	24,838	24,838	24,838	24,838

Notes: The sample is restricted to participants' first repeat reporting of their birth location. 95% confidence intervals based on heteroskedasticity robust standard errors are shown in brackets. Standard errors for $\hat{p}$ were computed using the delta method.

Supplementary Results 4: Attenuation bias

a Ordinary least squares

Variance share from time variation

Spatial autocorrelation (ρ)	0%	20%	40%	60%	80%	100%
0.000	27.9	22.4	16.9	11.3	5.7	0.0
0.050	28.5	22.8	17.1	11.4	5.7	0.0
0.100	27.6	22.1	16.6	11.0	5.5	0.0
0.150	28.2	22.6	16.9	11.3	5.6	0.0
0.200	27.9	22.3	16.7	11.1	5.6	0.0
0.250	27.5	21.9	16.4	11.0	5.5	0.0
0.300	27.6	22.1	16.6	11.1	5.5	0.0
0.350	27.2	21.8	16.4	11.0	5.5	0.0
0.400	27.3	21.8	16.4	11.0	5.5	0.0
0.450	27.5	22.0	16.5	11.0	5.5	0.0
0.500	27.4	22.0	16.5	11.0	5.5	0.0
0.550	27.2	21.7	16.2	10.8	5.4	0.0
0.600	26.4	21.1	15.8	10.5	5.2	0.0
0.650	26.7	21.4	16.1	10.7	5.4	0.0
0.700	26.1	20.9	15.7	10.4	5.2	0.0
0.750	25.1	20.1	15.1	10.0	5.0	0.0
0.800	25.4	20.4	15.4	10.3	5.2	0.0
0.850	24.7	19.6	14.7	9.8	4.9	0.0
0.900	22.8	18.3	13.8	9.3	4.7	0.0
0.950	20.3	16.2	12.2	8.1	4.0	0.0
0.975	17.0	13.7	10.3	7.0	3.5	0.0

b Sibling fixes effects

Variance share from time variation

Spatial autocorrelation (ρ)	0%	20%	40%	60%	80%	100%
0.000	90.1	61.5	40.3	23.8	10.7	0.0
0.050	89.7	61.9	40.8	24.3	11.0	0.0
0.100	90.1	61.4	40.1	23.7	10.7	0.0
0.150	89.8	61.8	40.7	24.1	10.9	0.0
0.200	89.9	61.8	40.6	24.2	10.9	0.0
0.250	90.1	61.2	40.0	23.6	10.6	0.0
0.300	90.1	61.3	40.0	23.5	10.5	0.0
0.350	90.1	61.3	40.1	23.7	10.7	0.0
0.400	90.1	61.4	40.1	23.6	10.6	0.0
0.450	90.0	61.7	40.6	24.1	10.9	0.0
0.500	90.0	61.8	40.5	24.0	10.8	0.0
0.550	89.9	61.6	40.3	23.9	10.7	0.0
0.600	90.1	61.0	39.6	23.3	10.4	0.0
0.650	89.9	60.9	39.6	23.3	10.5	0.0
0.700	90.0	61.0	39.7	23.4	10.5	0.0
0.750	89.9	59.6	38.3	22.3	9.9	0.0
0.800	89.9	60.2	39.0	22.9	10.2	0.0
0.850	90.0	59.3	37.9	22.0	9.7	0.0
0.900	90.1	58.3	36.8	21.2	9.4	0.0
0.950	89.9	56.0	34.4	19.4	8.4	0.0
0.975	89.8	52.2	30.9	17.0	7.3	0.0

Supplementary Figure 4: Attenuation bias for coordinate-level data with different levels of spatial correlation and time variation. Panel **a** shows the bias in ordinary least squares estimations, panel **b** the bias in sibling fixed effects estimation. The attenuation bias values shown are the mean bias (in %) from simulations of OLS and sibling fixed effects estimations with $r = 1000$ repetitions and sample sizes of $n = 36,762$ individuals each. For each level of spatial autocorrelation (ρ), 10 coordinate-level variables were simulated at a 1km resolution and merged to the sibling sample. The coordinate-level spatial variables were combined with normally distributed year-month of birth fixed effects to simulate time-varying spatial exposures. The columns of the tables correspond to different ratios of spatial to temporal variation when simulating the exposure variable - as indicated by the share of the exposure variance due to time variation. Each simulated variable was then used in 100 simulations of the attenuation bias based on an error probability for the location of birth $p = 0.284$ and a move probability $q = 0.012$. Source data are provided as a source data file.

a Ordinary least squares

Variance share from time variation

Spatial autocorrelation (ρ)	0%	20%	40%	60%	80%	100%
**0.000**	17.0	13.6	10.2	6.8	3.4	0.0
**0.050**	16.6	13.3	10.0	6.7	3.4	0.0
**0.100**	16.5	13.2	9.9	6.6	3.3	0.0
**0.150**	16.6	13.2	9.9	6.6	3.3	0.0
**0.200**	15.7	12.5	9.4	6.2	3.1	0.0
**0.250**	15.4	12.3	9.3	6.2	3.1	0.0
**0.300**	16.0	12.8	9.7	6.5	3.3	0.0
**0.350**	15.9	12.7	9.5	6.3	3.2	0.0
**0.400**	16.1	12.9	9.7	6.5	3.3	0.0
**0.450**	15.8	12.6	9.5	6.3	3.2	0.0
**0.500**	15.7	12.5	9.4	6.2	3.1	0.0
**0.550**	15.4	12.3	9.3	6.2	3.1	0.0
**0.600**	15.5	12.4	9.3	6.2	3.1	0.0
**0.650**	14.9	12.0	9.0	6.0	3.0	0.0
**0.700**	14.3	11.4	8.6	5.7	2.8	0.0
**0.750**	14.6	11.7	8.8	5.9	2.9	0.0
**0.800**	13.4	10.7	8.0	5.4	2.7	0.0
**0.850**	13.3	10.6	8.0	5.3	2.7	0.0
**0.900**	12.7	10.1	7.6	5.0	2.5	0.0
**0.950**	11.4	9.1	6.9	4.6	2.3	0.0
**0.975**	10.8	8.6	6.4	4.3	2.1	0.0

b Sibling fixes effects

Variance share from time variation

Spatial autocorrelation (ρ)	0%	20%	40%	60%	80%	100%
**0.000**	88.4	52.2	31.1	17.2	7.4	0.0
**0.050**	88.2	52.0	31.0	17.1	7.3	0.0
**0.100**	88.3	52.1	31.0	17.2	7.4	0.0
**0.150**	88.1	52.4	31.4	17.4	7.5	0.0
**0.200**	88.4	50.8	29.7	16.2	6.9	0.0
**0.250**	88.3	50.3	29.3	15.9	6.7	0.0
**0.300**	88.2	51.5	30.5	16.8	7.2	0.0
**0.350**	88.2	51.2	30.1	16.5	7.0	0.0
**0.400**	88.3	51.4	30.4	16.8	7.2	0.0
**0.450**	88.2	50.9	29.9	16.4	7.0	0.0
**0.500**	88.4	50.9	30.0	16.4	7.0	0.0
**0.550**	88.3	50.2	29.3	16.0	6.8	0.0
**0.600**	88.3	50.5	29.6	16.2	6.9	0.0
**0.650**	88.3	49.7	28.7	15.6	6.5	0.0
**0.700**	87.9	48.8	28.0	15.2	6.4	0.0
**0.750**	88.3	49.3	28.4	15.3	6.4	0.0
**0.800**	88.2	46.8	26.3	14.1	5.9	0.0
**0.850**	88.2	47.2	26.6	14.2	5.9	0.0
**0.900**	88.2	46.0	25.6	13.6	5.6	0.0
**0.950**	88.0	43.4	23.6	12.4	5.1	0.0
**0.975**	88.3	42.8	23.2	12.1	4.9	0.0

Supplementary Figure 5: Attenuation bias for parish-level data with different levels of spatial correlation and time variation. Panel **a** shows the bias in ordinary least squares estimations, panel **b** the bias in sibling fixed effects estimation. The attenuation bias values shown are the mean bias (in %) from simulations of OLS and sibling fixed effects estimations with $r = 1000$ repetitions and sample sizes of $n = 36,950$ individuals each. For each level of spatial autocorrelation (ρ), 10 parish-level variables were simulated and merged to the sibling sample. The parish-level spatial variables were combined with normally distributed year-month of birth fixed effects to simulate time-varying spatial exposures. The columns of the tables correspond to different ratios of spatial to temporal variation when simulating the exposure variable - as indicated by the share of the exposure variance due to time variation. Each simulated variable was then used in 100 simulations of the attenuation bias based on an error probability for the parish of birth $p = 0.168$ and a move probability $q = 0.009$. Source data are provided as a source data file.

Supplementary Results 5: Bias in analyses controlling for district of birth fixed effects

Correlation of X and fixed effects

Spatial autocorrelation (ρ)	-0.95	-0.75	-0.50	-0.25	0.00	0.25	0.50	0.75	0.95
0.000	-0.74	-0.34	-0.16	-0.07	0.00	0.07	0.16	0.33	0.74
0.050	-0.74	-0.34	-0.16	-0.07	0.00	0.07	0.16	0.34	0.74
0.100	-0.75	-0.34	-0.16	-0.07	0.00	0.07	0.16	0.34	0.75
0.150	-0.74	-0.33	-0.16	-0.07	0.00	0.07	0.16	0.33	0.74
0.200	-0.74	-0.33	-0.16	-0.07	0.00	0.07	0.16	0.33	0.74
0.250	-0.73	-0.32	-0.15	-0.07	0.00	0.07	0.15	0.33	0.74
0.300	-0.74	-0.33	-0.16	-0.07	0.00	0.07	0.16	0.33	0.74
0.350	-0.74	-0.33	-0.16	-0.07	0.00	0.07	0.16	0.33	0.74
0.400	-0.73	-0.32	-0.15	-0.06	0.00	0.06	0.15	0.32	0.73
0.450	-0.73	-0.32	-0.15	-0.06	0.00	0.06	0.15	0.32	0.73
0.500	-0.72	-0.31	-0.14	-0.06	0.00	0.06	0.15	0.31	0.72
0.550	-0.71	-0.30	-0.14	-0.06	0.00	0.06	0.14	0.30	0.72
0.600	-0.70	-0.29	-0.13	-0.06	0.00	0.06	0.13	0.29	0.69
0.650	-0.70	-0.28	-0.13	-0.05	0.00	0.06	0.13	0.28	0.69
0.700	-0.70	-0.29	-0.13	-0.06	0.00	0.06	0.13	0.29	0.70
0.750	-0.69	-0.27	-0.13	-0.05	0.00	0.05	0.13	0.27	0.68
0.800	-0.68	-0.27	-0.12	-0.05	0.00	0.05	0.12	0.27	0.68
0.850	-0.64	-0.24	-0.11	-0.05	0.00	0.05	0.11	0.24	0.65
0.900	-0.62	-0.22	-0.10	-0.04	0.00	0.04	0.10	0.22	0.62
0.950	-0.59	-0.20	-0.09	-0.04	0.00	0.04	0.09	0.20	0.59
0.975	-0.55	-0.18	-0.08	-0.03	0.00	0.03	0.08	0.18	0.55

Supplementary Figure 6: Bias when controlling for district of birth fixed effects. The bias values shown are the mean bias for the coefficient of interest when district of birth fixed effects are included as control variables. The mean bias is expressed in units of σ_μ/σ_X and was derived in simulations of OLS estimations with 1000 repetitions. The individual sample size in the simulations is $n = 36,940$, with the number of singleton observations (i.e. individuals with a unique district of birth) dropped in the estimations differing across repetitions. For each level of spatial autocorrelation (ρ), 10 district-level fixed effects were simulated and merged to the sibling sample. Each fixed effects variable was then used in 100 simulations of the bias (25 each for $\sigma_\mu/\sigma_x \in [0.1, 0.5, 1.0, 5.0]$) based on an error probability for the district of birth $p = 0.158$ and a move probability $q = 0.009$. The columns of the tables correspond to different correlations between the variable of interest X and the district of birth fixed effects. The rows of the tables correspond to different spatial autocorrelations ρ of the district of birth fixed effects. Source data are provided as a source data file.

Supplementary Results 6: Spatial distribution of genetic principal components

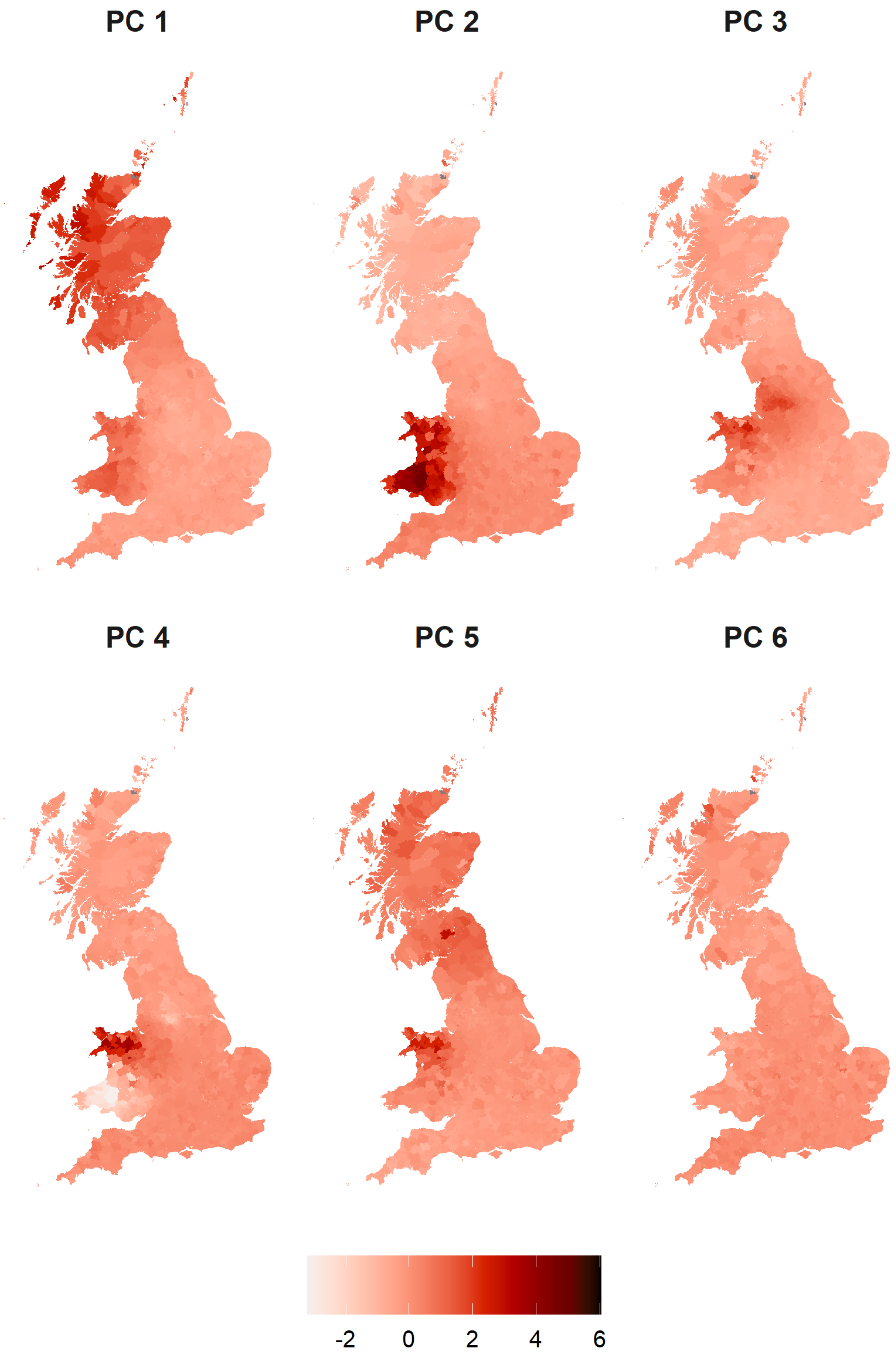

Supplementary Figure 7: Geographical distribution of genetic principal components. The figures shows for each district the mean genetic principal components of UKB participants who reported being born in this district. The figures are based on a sample size of $n = 310,020$ individuals. The district boundary map is from www.VisionofBritain.org.uk[6] and is copyright of the Great Britain Historical GIS Project and the University of Portsmouth.

Supplementary Analysis 1: Phenotype comparisons in Abdellaoui et al. (2019)

In the following, we examine the relevance of potentially mismeasured birth location data in the UK Biobank for comparisons of individuals born in coal-mining and non-coal mining areas, following Abdellaoui et al. (2019) [1]. They use the birth location and current home location of UKB participants to determine whether they were born in a coal-mining area or not and whether they migrated into / out of coal-mining areas (or stayed in the type of area they were born in). Measurement error in the birth location data will thus affect the correct assignment of participants to these groups. Estimations similar to those reported in Table 1 allow us to quantify the probability of incorrect assignment of a participant to birth in a coal-mining area or a non-coal area. We estimate that this assignment is incorrect with a probability of $\widehat{p} = 3.9\%$. We furthermore estimate the annual probability of household moves from coal mining to non-coal mining area or vice versa to be 0.3% (between the births of UKB sibling pairs).

In Supplementary Figure 8 we replicate comparisons of five phenotypes between those born in coal mining areas and those born elsewhere in Great Britain (replication of Supplementary Figure 9 in Abdellaoui et al., 2019). We conduct these replications in two different sub-samples of the UK Biobank. First, in the sample of sibling pairs used in our main analyses (restricted to the two oldest siblings per family with birthplace in England / Wales / Scotland), with the corresponding phenotype means shown in blue. Second, we restrict this sample to sibling pairs which were either both born in coal mining areas or both born elsewhere (we refer to this as the "robust" sibling sample), with the corresponding phenotype means shown in red. For the "robust" sub-sample we ensure siblings' location of birth matches at the geographical level of variation used; in this case coal versus non-coal areas. This sensitivity analysis allows us to limit the sample to individuals with more reliable birth location information. While the mean differences between the two samples are not large, there is a clear tendency across all five phenotypes: the differences between those born in coal-mining areas and those born elsewhere is consistently larger in the "robust" sub-sample. This is most noticeable in the case of educational attainment, with the gap increasing from 0.25 to 0.28 standard deviations when dropping sibling pairs with less reliable birth location information. This suggests that the two samples differ, with the "robust" sub-sample showing a wider variation in phenotypes. Not taking this into account therefore underestimates the differences between coal-mining and non-coal areas.

In Supplementary Figure 9 we replicate comparisons of phenotypes between four migration groups from Figure 4 in Abdellaoui et al. (2019). These groups are: (1) those who moved from a coal mining area to a non-coal area; (2) those who stayed in a non-coal area; (3) those who moved from a non-coal area to coal

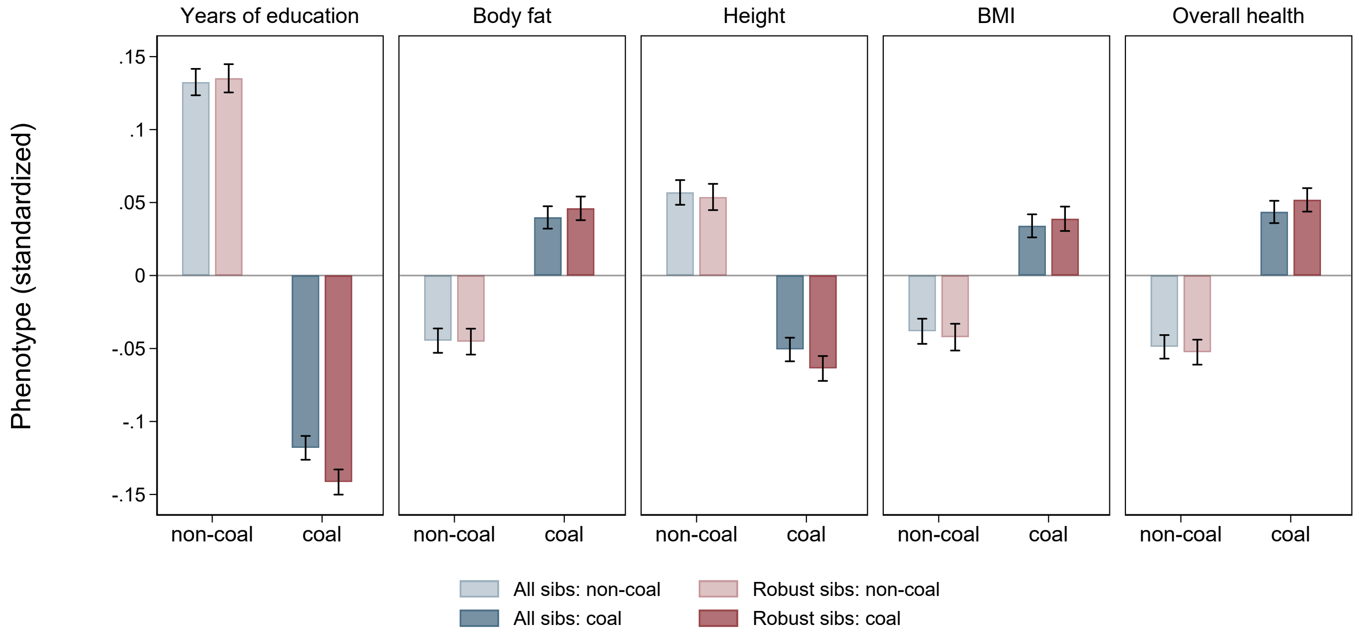

Supplementary Figure 8: Phenotype differences between coal mining regions and the rest of Great Britain - sibling sensitivity analysis. The figure shows the means of standardized phenotypes for those born in coal mining areas and for those born elsewhere. Means were calculated for two sub-samples of the UK Biobank: All sibs – the sample of siblings in our main analysis (two oldest siblings per family with birthplace in England / Wales / Scotland); Robust sibs – only sibling pairs which were both born in a coal area or both born in non-coal areas. Vertical bars represent standard errors. Source data including the sample sizes for each phenotype and sub-sample are provided as a source data file.

mining area; (4) those who stayed in a coal mining area. Again, we estimate phenotypes for two sub-samples, the sibling sample from our main analyses (in blue) and a sample of sibling pairs with robust birth location data (in red). Overall, the phenotype means in the two samples are similar. The most noticeable differences are for those moving into coal-mining areas. In the "robust" sibling sample the mean educational attainment, body fat, BMI and overall health of this group is more similar to those moving away from coal-mining areas. Indeed, the gap in years of education between the two mover groups is left insignificant in the "robust" sample. Again, this suggests that the phenotypes differ across the two sub-samples.

While our findings from these sensitivity analyses do not alter the main conclusions of the study by Abdellaoui et al. (2019), they illustrate that mismeasured birth locations can have sizeable and meaningful consequences.

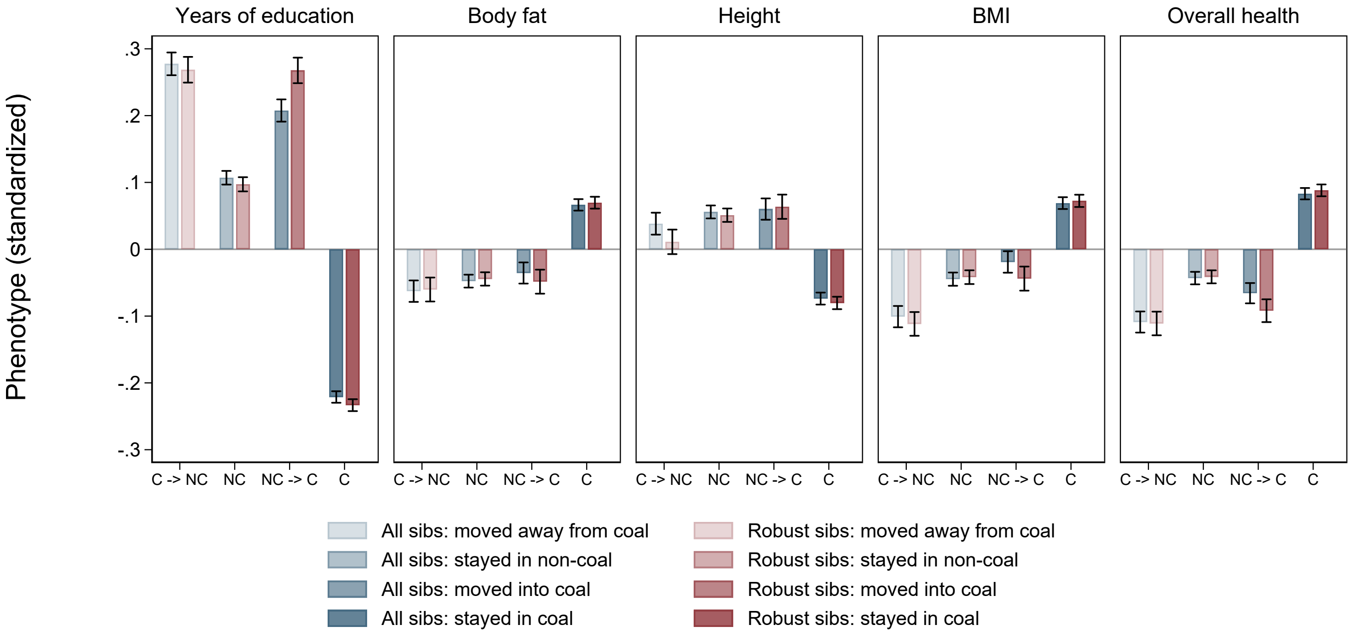

Supplementary Figure 9: Phenotype differences between migration groups - sibling sensitivity analysis. The figure shows the means of standardized phenotypes for four migration groups defined based on their reported birth locations and their home location in 2006-10: (1) moved from coal area to non-coal area; (2) stayed in non-coal area; (3) moved from non-coal area to coal area; (4) stayed in coal area. Means were calculated for two sub-samples of the UK Biobank: All sibs – the sample of siblings in our main analysis (two oldest siblings per family with birthplace in England / Wales / Scotland); Robust sibs – only sibling pairs which were both born in a coal area or both born in non-coal areas. Vertical bars represent standard errors. Source data including the sample sizes for each phenotype and migration group are provided as a source data file.

Supplementary Results 7: Descriptive statistics

Supplementary Table 11: Descriptive statistics

	Mean	SD	Min	Max	N
**Individual characteristics:**					
Female	0.58	0.49	0.00	1.00	36,958
Year of birth	1950.99	7.36	1937.00	1970.00	36,958
Years of education	12.92	2.30	10.00	16.00	36,695
Degree qualification	0.29	0.45	0.00	1.00	36,695
Upper secondary qualification	0.63	0.48	0.00	1.00	36,695
**Family characteristics:**					
Age gap (years)	4.53	3.19	0.00	27.67	18,479
Mean PGI for education	0.00	1.00	−3.79	4.14	18,045
**District of birth characteristics:**					
Rural district	0.10	0.31	0.00	1.00	33,862
Urban / municipal district	0.86	0.34	0.00	1.00	33,862
Metropolitan district	0.03	0.17	0.00	1.00	33,862
Average population density 1940-70	3246.56	2453.58	5.28	20555.02	33,862
Share left FT education by 14 (1951)	0.77	0.07	0.41	0.90	33,862
**Birth location differences:**					
Distance (km)	21.67	68.56	0.00	1060.39	18,479
Different parish	0.35	0.48	0.00	1.00	18,479
Different district	0.33	0.47	0.00	1.00	18,479
Different county	0.15	0.36	0.00	1.00	18,479
Sibling distance > 0km	0.54	0.50	0.00	1.00	18,479
Sibling distance > 5km	0.30	0.46	0.00	1.00	18,479
Sibling distance > 10km	0.19	0.39	0.00	1.00	18,479
Sibling distance > 20km	0.14	0.34	0.00	1.00	18,479
Sibling distance > 30km	0.12	0.32	0.00	1.00	18,479
Sibling distance > 50km	0.09	0.29	0.00	1.00	18,479

Notes: The table shows the mean, standard deviation (SD), minimum value (Min), maximum value (Max) and number of observations (N) for individual, family and birth location characteristics of our analysis sample. Descriptive statistics for individual and district of birth characteristics were obtained at the individual level. Descriptive statistics for family characteristics and birth location differences were obtained at the family level.

Supplementary Table 12: Comparison of sibling sample and full UK Biobank sample

	Sibling sample			Full UKB sample		
	Mean	SD	N	Mean	SD	N
**Individual characteristics:**						
Female	0.58	0.49	36,958	0.54	0.50	502,336
Year of birth	1950.99	7.36	36,958	1951.54	8.12	502,336
Years of education	12.92	2.30	36,695	13.11	2.33	493,602
Degree qualification	0.29	0.45	36,695	0.33	0.47	493,602
Upper secondary qualification	0.63	0.48	36,695	0.66	0.47	493,602
**District of birth characteristics:**						
Rural district	0.10	0.31	33,862	0.11	0.32	405,088
Urban / municipal district	0.86	0.34	33,862	0.84	0.37	405,088
Metropolitan district	0.03	0.17	33,862	0.05	0.22	405,088
Average population density 1940-70	3246.56	2453.58	33,862	3217.94	2752.17	405,088
Share left FT education by 14 (1951)	0.77	0.07	33,862	0.76	0.08	405,088

Notes: The table compares the mean, standard deviation (SD) and number of observations (N) for individual and district of birth characteristics between the sibling sample used in our analysis and the full UK Biobank (UKB) sample.

Supplementary Note 2: Attenuation bias - Classical measurement error model

In the following, we use the case of classical measurement error as an illustration of the attenuation bias. Consider the bivariate linear model:

$$Y = \alpha + \beta X + \varepsilon \tag{1}$$

where Y denotes the outcome of interest, X is individuals' early life environment, and ε is an idiosyncratic error term. Since we cannot observe X directly, we use a measure based on individuals' reported date and place of birth. This measure of early life environment X^* is subject to measurement error u due to reporting / recording errors of the birth place as well as due to household moves during childhood:

$$X^* = X + u \tag{2}$$

Estimating Equation 1 using the observable measure X^* instead of X leads to attenuation bias in $\hat{\beta}$ due to the measurement error:

$$\begin{aligned} \hat{\beta} = \frac{Cov(Y, X^*)}{Var(X^*)} &= \frac{Cov(\alpha + \beta X + \varepsilon, X + u)}{Var(X + u)} = \frac{\beta\,[Var(X) + Cov(X, u)]}{Var(X) + Var(u) + 2Cov(X, u)} \\ &= \left[1 - \frac{Var(u) + Cov(X, u)}{Var(X) + Var(u) + 2Cov(X, u)}\right]\beta = \left[1 - \frac{Cov(X^*, u)}{Var(X^*)}\right]\beta = [1 - \lambda]\beta \end{aligned} \tag{3}$$

where λ is the magnitude of the attenuation bias. λ is increasing in the variance of the measurement error, which is driven (i) by the probability of an error in the birth location, (ii) by the difference in the exposure between the true and the reported place of birth (i.e., X and X^*), and (iii) by the share of the variation in the exposure that is driven by temporal (vs spatial) variation.

Supplementary Results 8: Distribution of measurement error

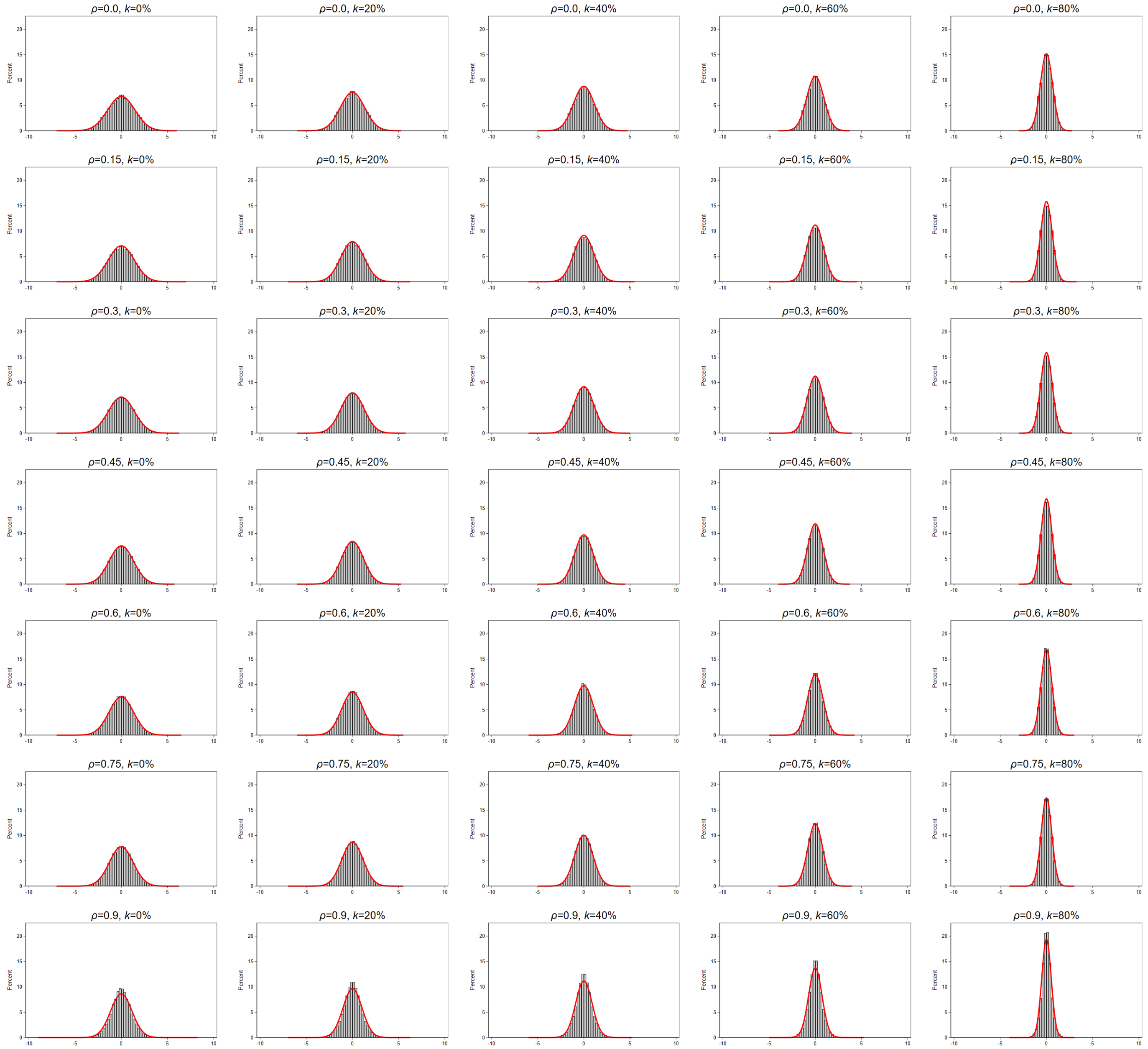

Supplementary Figure 10: Distribution of non-zero errors for simulated district-level data. The figures show a histogram of the non-zero measurement errors in district-level simulations for different levels of autocorrelation ρ and variance shares from time variation k. For comparison, a normal distribution of the same mean and standard deviation is overlaid in red. Each histogram is based on $r = 100$ repetitions with samples of $n = 36,940$ individuals each (prior to the exclusion of observations with zero error). For district-level variables, approximately $1 - \widehat{p} = 84\%$ of errors are zero. There is no measurement error when $k = 100\%$, hence this case is omitted here.

Supplementary Table 13: Correlations of measurement error and true variable for simulated district-level data

	Variance share from time variation (k)				
Spatial autocorrelation (ρ)	0%	20%	40%	60%	80%
$\rho = 0.00$	-0.287<br>[-0.288,-0.286]	-0.256<br>[-0.257,-0.255]	-0.222<br>[-0.223,-0.221]	-0.180<br>[-0.181,-0.179]	-0.127<br>[-0.128,-0.126]
$\rho = 0.15$	-0.274<br>[-0.275,-0.273]	-0.245<br>[-0.246,-0.244]	-0.213<br>[-0.214,-0.212]	-0.174<br>[-0.175,-0.173]	-0.123<br>[-0.124,-0.122]
$\rho = 0.30$	-0.273<br>[-0.274,-0.272]	-0.245<br>[-0.246,-0.244]	-0.212<br>[-0.213,-0.211]	-0.172<br>[-0.173,-0.171]	-0.122<br>[-0.123,-0.121]
$\rho = 0.45$	-0.257<br>[-0.258,-0.256]	-0.231<br>[-0.232,-0.230]	-0.200<br>[-0.201,-0.199]	-0.164<br>[-0.165,-0.163]	-0.116<br>[-0.117,-0.115]
$\rho = 0.60$	-0.257<br>[-0.258,-0.256]	-0.231<br>[-0.232,-0.230]	-0.200<br>[-0.201,-0.199]	-0.163<br>[-0.164,-0.162]	-0.115<br>[-0.117,-0.114]
$\rho = 0.75$	-0.250<br>[-0.251,-0.249]	-0.224<br>[-0.225,-0.223]	-0.194<br>[-0.195,-0.193]	-0.159<br>[-0.160,-0.158]	-0.113<br>[-0.114,-0.112]
$\rho = 0.90$	-0.225<br>[-0.226,-0.224]	-0.200<br>[-0.201,-0.199]	-0.173<br>[-0.174,-0.172]	-0.140<br>[-0.141,-0.139]	-0.099<br>[-0.100,-0.098]

Notes: The table shows correlations between the true variable X and the measurement error u in district-level simulations for different levels of autocorrelation ρ and variance shares from time variation k. Each correlation is based on $r = 100$ repetitions with samples of $n = 36,940$ individuals each. For each level of spatial autocorrelation (ρ), 10 district-level variables were simulated and merged to the sibling sample. The district-level spatial variables were combined with normally distributed year-month of birth fixed effects to simulate time-varying spatial exposures. The columns of the table correspond to different ratios of spatial to temporal variation when simulating the exposure variable - as indicated by the share of the exposure variance due to time variation. Each simulated variable was then used in 10 simulations of the measurement error and the true variable based on an error probability for the district of birth $p = 0.158$ and a move probability $q = 0.009$. There is no measurement error when $k = 100\%$, hence this case is omitted here.

Supplementary Table 14: Correlations of measurement error and true variable for simulated district-level data - observations with non-zero measurement error only

	Variance share from time variation (k)				
Spatial autocorrelation (ρ)	0%	20%	40%	60%	80%
$\rho = 0.00$	-0.710<br>[-0.711,-0.708]	-0.637<br>[-0.638,-0.635]	-0.556<br>[-0.558,-0.554]	-0.455<br>[-0.457,-0.453]	-0.324<br>[-0.326,-0.321]
$\rho = 0.15$	-0.692<br>[-0.693,-0.690]	-0.620<br>[-0.622,-0.619]	-0.540<br>[-0.542,-0.538]	-0.442<br>[-0.444,-0.439]	-0.314<br>[-0.316,-0.311]
$\rho = 0.30$	-0.683<br>[-0.685,-0.682]	-0.614<br>[-0.616,-0.612]	-0.535<br>[-0.537,-0.533]	-0.437<br>[-0.439,-0.435]	-0.311<br>[-0.313,-0.309]
$\rho = 0.45$	-0.661<br>[-0.662,-0.659]	-0.591<br>[-0.593,-0.589]	-0.511<br>[-0.513,-0.509]	-0.420<br>[-0.422,-0.418]	-0.297<br>[-0.299,-0.295]
$\rho = 0.60$	-0.651<br>[-0.653,-0.650]	-0.586<br>[-0.588,-0.584]	-0.508<br>[-0.510,-0.506]	-0.417<br>[-0.419,-0.414]	-0.295<br>[-0.297,-0.293]
$\rho = 0.75$	-0.618<br>[-0.620,-0.617]	-0.558<br>[-0.560,-0.556]	-0.487<br>[-0.489,-0.485]	-0.400<br>[-0.402,-0.398]	-0.286<br>[-0.288,-0.284]
$\rho = 0.90$	-0.558<br>[-0.560,-0.557]	-0.501<br>[-0.503,-0.499]	-0.436<br>[-0.438,-0.434]	-0.356<br>[-0.358,-0.353]	-0.252<br>[-0.255,-0.250]

Notes: The table shows correlations between the true variable X and the measurement error u in district-level simulations for different levels of autocorrelation ρ and variance shares from time variation k. Correlations are calculated for observations with non-zero measurement error only ($u \neq 0$). Each correlation is based on $r = 100$ repetitions with samples of $n = 36,940$ individuals each (prior to the exclusion of observations with zero error). For each level of spatial autocorrelation (ρ), 10 district-level variables were simulated and merged to the sibling sample. The district-level spatial variables were combined with normally distributed year-month of birth fixed effects to simulate time-varying spatial exposures. The columns of the table correspond to different ratios of spatial to temporal variation when simulating the exposure variable - as indicated by the share of the exposure variance due to time variation. Each simulated variable was then used in 10 simulations of the measurement error and the true variable based on an error probability for the district of birth $p = 0.158$ and a move probability $q = 0.009$. There is no measurement error when $k = 100\%$, hence this case is omitted here.

Supplementary References

[1] Abdellaoui, A. *et al.* Genetic correlates of social stratification in Great Britain. *Nature Human Behaviour* **3**, 1332–1342 (2019).

[2] Haworth, S. *et al.* Apparent latent structure within the UK Biobank sample has implications for epidemiological analysis. *Nature Communications* **10**, 333 (2019).

[3] Howe, L. J. *et al.* Genetic evidence for assortative mating on alcohol consumption in the UK Biobank. *Nature Communications* **10**, 5039 (2019).

[4] von Hinke, S. & Sørensen, E. N. The long-term effects of early-life pollution exposure: Evidence from the London Smog. *Journal of Health Economics* **92**, 102827 (2023).

[5] General Register Office. *Census 1951: England and Wales: County Reports* (Her Majesty's Stationery Office, 1955).

[6] Great Britain Historical GIS Project. A Vision of Britain through Time (2017). URL: https://www.visionofbritain.org.uk.

[7] Becker, J. *et al.* Resource profile and user guide of the Polygenic Index Repository. *Nature Human Behaviour* **5**, 1744–1758 (2021).
