# Supplementary material for: An analysis of the accuracy of retrospective birth location recall using sibling data
Source: Nat Commun. 2024 Mar 26;15:2665. doi: 10.1038/s41467-024-46781-z (PMC10965954; doi:10.1038/s41467-024-46781-z)
Supplement: Supplementary file 3 — Reporting Summary [file 41467_2024_46781_MOESM3_ESM.pdf]

nature portfolio

Corresponding author(s): Stephanie von Hinke, Nicolai Vitt

Last updated by author(s): Feb 12, 2024

Reporting Summary

Nature Portfolio wishes to improve the reproducibility of the work that we publish. This form provides structure for consistency and transparency in reporting. For further information on Nature Portfolio policies, see our Editorial Policies and the Editorial Policy Checklist.

Statistics

For all statistical analyses, confirm that the following items are present in the figure legend, table legend, main text, or Methods section.

n/a	Confirmed	
☐	☒	The exact sample size (*n*) for each experimental group/condition, given as a discrete number and unit of measurement
☐	☒	A statement on whether measurements were taken from distinct samples or whether the same sample was measured repeatedly
☐	☒	The statistical test(s) used AND whether they are one- or two-sided<br>*Only common tests should be described solely by name; describe more complex techniques in the Methods section.*
☐	☒	A description of all covariates tested
☐	☒	A description of any assumptions or corrections, such as tests of normality and adjustment for multiple comparisons
☐	☒	A full description of the statistical parameters including central tendency (e.g. means) or other basic estimates (e.g. regression coefficient) AND variation (e.g. standard deviation) or associated estimates of uncertainty (e.g. confidence intervals)
☐	☒	For null hypothesis testing, the test statistic (e.g. *F*, *t*, *r*) with confidence intervals, effect sizes, degrees of freedom and *P* value noted<br>*Give P values as exact values whenever suitable.*
☒	☐	For Bayesian analysis, information on the choice of priors and Markov chain Monte Carlo settings
☒	☐	For hierarchical and complex designs, identification of the appropriate level for tests and full reporting of outcomes
☒	☐	Estimates of effect sizes (e.g. Cohen's *d*, Pearson's *r*), indicating how they were calculated

Our web collection on statistics for biologists contains articles on many of the points above.

Software and code

Policy information about availability of computer code

Data collection	Data was collected by the UK Biobank. Details of the data collection are described at Bycroft et al. (2018), The UK Biobank resource with deep phenotyping and genomic data. Nature 562, 203–209.
Data analysis	Data was analysed in Stata version 17 and R version 4.2.1. The code is available under https://doi.org/10.5281/zenodo.10631529

For manuscripts utilizing custom algorithms or software that are central to the research but not yet described in published literature, software must be made available to editors and reviewers. We strongly encourage code deposition in a community repository (e.g. GitHub). See the Nature Portfolio guidelines for submitting code & software for further information.

Data

Policy information about availability of data

All manuscripts must include a data availability statement. This statement should provide the following information, where applicable:

- Accession codes, unique identifiers, or web links for publicly available datasets
- A description of any restrictions on data availability
- For clinical datasets or third party data, please ensure that the statement adheres to our policy

This research has been conducted using data from UK Biobank, a major biomedical database (Project ID: 74002). UK Biobank data are available following an application procedure described at https://www.ukbiobank.ac.uk/enable-your-research.

This research is furthermore based on data provided through www.VisionofBritain.org.uk and uses historical material which is copyright of the Great Britain Historical GIS Project and the University of Portsmouth. Data on boundaries of historic parishes, districts and counties in 1951 have previously been made available by the Vision of Britain project. For details on the current or future availability of the boundary data, please see https://www.visionofbritain.org.uk/data/. District-level demographic data from the Registrar General's Statistical Review of England and Wales are available via the UK data service at https://doi.org/10.5255/UKDA-SN-9035-1. District-level data on housing density, social class and education from the 1951 census are available via the UK data service at https://doi.org/10.5255/UKDA-SN-4554-2, https://doi.org/10.5255/UKDA-SN-4561-2 and https://doi.org/10.5255/UKDA-SN-4552-2. Disease data are from the Registrar General's Weekly Reports.

Source data for figures are provided with this paper.

Research involving human participants, their data, or biological material

Policy information about studies with human participants or human data. See also policy information about sex, gender (identity/presentation), and sexual orientation and race, ethnicity and racism.

Reporting on sex and gender	We use the term sex to refer to the biological attribute. Our main analysis does not stratify the sample by sex, but in the heterogeneity analysis we conduct an analysis stratifying by the sex composition of the sibling pairs (based on self-reported sex in the UK Biobank).
Reporting on race, ethnicity, or other socially relevant groupings	Our main analyses do not control for or stratify by race or ethnicity. In line with the current genetics literature, we restrict our sample in estimations involving polygenic indices to those of European ancestry. In the principal component analysis, we restrict our sample to white-British individuals and exclude any genetic outliers.
Population characteristics	The respondents in our sibling sub-sample of the UK Biobank are born between 1937 and 1970. Approximately 58% of the sample are female.
Recruitment	We use the UK Biobank, which is a self-selected sample. Our analysis focuses on the accuracy of the birth location data among this self-selected sample, and we are unable to accurately extrapolate to data with different recruitment approaches.
Ethics oversight	The UK Biobank has received ethical approval as a Research Tissue Bank (RTB) from the NHS North West Centre for Research Ethics Committees (references: 11/NW/0382, 16/NW/0274, 21/NW/0157). Analysis of this secondary dataset does not require separate ethical approval.

Note that full information on the approval of the study protocol must also be provided in the manuscript.

Field-specific reporting

Please select the one below that is the best fit for your research. If you are not sure, read the appropriate sections before making your selection.

☐ Life sciences ☒ Behavioural & social sciences ☐ Ecological, evolutionary & environmental sciences

For a reference copy of the document with all sections, see nature.com/documents/nr-reporting-summary-flat.pdf

Behavioural & social sciences study design

All studies must disclose on these points even when the disclosure is negative.

Study description	We conduct a quantitative analysis of the accuracy of retrospective birth location data in the UK Biobank. We use the sibling sample in the UK Biobank to quantify the probabilities of measurement error and household moves. Using simulations we show the consequences of these inaccuracies for the use of birth location data in research applications. We furthermore outline possible solutions.
Research sample	We use the UK Biobank dataset. We restrict the UK Biobank sample to full siblings born in England, Wales and Scotland, keeping the oldest two siblings observed in each family. Our analysis sample comprises 36,958 siblings from 18,479 families. The focus on full siblings is to allow us to compare their reported birth location data, thereby quantifying the probabilities of household moves and measurement error. The respondents are born between 1937 and 1970, and approx. 58% of the sample are female. The UK Biobank is not representative of the UK population such that women, healthy individuals and those from less deprived areas are over-represented in the sample (Fry et al., 2017. Comparison of sociodemographic and health-related characteristics of UK Biobank participants with those of the general population, American journal of epidemiology, 186 (9), 1026–1034).
Sampling strategy	We use secondary data from the UK Biobank. The UK Biobank contacted over 5 million residents of the UK by mail, with approximately 10% volunteering to participate. For further details on the sampling strategy see: Bycroft et al. (2018), The UK Biobank resource with deep phenotyping and genomic data. Nature 562, 203–209.
Data collection	We use secondary data from the UK Biobank. For further details on the collection of the data see: Bycroft et al. (2018), The UK Biobank resource with deep phenotyping and genomic data. Nature 562, 203–209.
Timing	The initial data collection for the UK Biobank took place between 2006 and 2010, with additional follow-up data collections taking place since then.
Data exclusions	We first restrict the UK Biobank sample to those identified as full siblings based on their genetic data, resulting in 41,441 participants.

Data exclusions	We then further restrict this sample to families with at least two siblings born in England, Wales and Scotland (3,388 participants dropped). Finally, we restrict the sample to the oldest two siblings observed in each family (1,095 participants dropped). Our final sample comprises 36,958 siblings from 18,479 families.
Non-participation	For details on (non-)participation in the UK Biobank see: Bycroft et al. (2018), The UK Biobank resource with deep phenotyping and genomic data. Nature 562, 203–209.
Randomization	No randomization was necessary for our analysis since the study aim was to describe a characteristic of the data (error in birth location data) rather than conduct causal inference. Our main analysis does not control for covariates, however in our supplementary analyses we stratify our analysis by birth cohorts, district types, population density, UKB assessment centre locations (at which the birth location was recorded), region of birth, siblings' sex, districts' socio-economic composition, and siblings' polygenic index (PGI) for education.

Reporting for specific materials, systems and methods

We require information from authors about some types of materials, experimental systems and methods used in many studies. Here, indicate whether each material, system or method listed is relevant to your study. If you are not sure if a list item applies to your research, read the appropriate section before selecting a response.

Materials & experimental systems

n/a	Involved in the study
☒	☐ Antibodies
☒	☐ Eukaryotic cell lines
☒	☐ Palaeontology and archaeology
☒	☐ Animals and other organisms
☒	☐ Clinical data
☒	☐ Dual use research of concern
☒	☐ Plants

Methods

n/a	Involved in the study
☒	☐ ChIP-seq
☒	☐ Flow cytometry
☒	☐ MRI-based neuroimaging

Plants

Seed stocks	*Report on the source of all seed stocks or other plant material used. If applicable, state the seed stock centre and catalogue number. If plant specimens were collected from the field, describe the collection location, date and sampling procedures.*
Novel plant genotypes	*Describe the methods by which all novel plant genotypes were produced. This includes those generated by transgenic approaches, gene editing, chemical/radiation-based mutagenesis and hybridization. For transgenic lines, describe the transformation method, the number of independent lines analyzed and the generation upon which experiments were performed. For gene-edited lines, describe the editor used, the endogenous sequence targeted for editing, the targeting guide RNA sequence (if applicable) and how the editor was applied.*
Authentication	*Describe any authentication procedures for each seed stock used or novel genotype generated. Describe any experiments used to assess the effect of a mutation and, where applicable, how potential secondary effects (e.g. second site T-DNA insertions, mosiacism, off-target gene editing) were examined.*
